# The endogenous opioid system in the medial prefrontal cortex mediates ketamine’s antidepressant-like actions

**DOI:** 10.21203/rs.3.rs-3190391/v1

**Published:** 2023-10-03

**Authors:** Christopher Pittenger, Cheng Jiang, Ralph DiLeone, Ronald Duman

**Affiliations:** Yale University School of Medicine; Yale University School of Medicine; Yale University; Department of Psychiatry, Yale School of Medicine

## Abstract

Recent studies have implicated the endogenous opioid system in the antidepressant actions of ketamine, but the underlying mechanisms remain unclear. We used a combination of pharmacological, behavioral, and molecular approaches in rats to test the contribution of the prefrontal endogenous opioid system to the antidepressant-like effects of a single dose of ketamine. Both the behavioral actions of ketamine and their molecular correlates in the medial prefrontal cortex (mPFC) were blocked by acute systemic administration of naltrexone, a competitive opioid receptor antagonist. Naltrexone delivered directly into the mPFC similarly disrupted the behavioral effects of ketamine. Ketamine treatment rapidly increased levels of β-endorphin and the expression of the μ-opioid receptor gene (*Oprm1*) in the mPFC, and the expression of the gene that encodes proopiomelanocortin, the precursor of β-endorphin, in the hypothalamus, *in vivo*. Finally, neutralization of β-endorphin in the mPFC using a specific antibody prior to ketamine treatment abolished both behavioral and molecular effects. Together, these findings indicate that presence of β-endorphin and activation of opioid receptors in the mPFC are required for the antidepressant-like actions of ketamine.

## INTRODUCTION

Ketamine, a noncompetitive antagonist of *N*-methyl-D-aspartate glutamate receptors (NMDARs), has been found to have rapid antidepressant and anti-suicide effects in many patients, and similar rapid antidepressant-like effects in preclinical models [1, 2]. In addition to its well-established ability to block NMDARs, ketamine also interacts with a range of other targets, including opioid receptors [3]. The endogenous opioid system consists of the μ-, δ-, and κ- opioid receptors (ORs), encoded by the *Oprm1*, *Oprd1*, and *Oprk1* genes; their primary endogenous ligands are the opioid peptides β-endorphin, enkephalins, and dynorphins, respectively. These receptors and peptides are implicated in a wide range of physiological and pathological processes, including the regulation of pain and of reward and stress responses [4–6]. Accumulating evidence implicates the endogenous opioid system in the pathophysiology and treatment of depression [7]. In particular, β-endorphin is implicated in both MDD and its treatment [8], and the μ-OR differs between healthy controls and patients with MDD in multiple brain regions [9, 10]. Previous preclinical studies extensively documented the role of medial prefrontal cortex (mPFC) in regulating ketamine’s antidepressant-like effects, implicating synaptic protein synthesis in the mPFC in ketamine’s actions [11, 12]. However, no study to date has examined the involvement of endogenous opioid signaling in the mPFC in ketamine’s behavioral and molecular effects.

In a seminal clinical study, Williams et al. reported that pretreatment with naltrexone, an opioid receptor antagonist, attenuates the antidepressant and anti-suicidal effects of ketamine in depressed patients [13, 14] (although another clinical study did not show such an effect in patients treated with naltrexone for alcohol use [15]). In preclinical work, systemic naltrexone pretreatment was found to block the antidepressant-like effects of ketamine in three studies [16–18], though not in a fourth [19]. Here, we test the hypothesis that β-endorphin and activation of opioid receptors in the mPFC is required for the antidepressant-like actions of ketamine, and for the underlying molecular mechanisms in the mPFC.

## MATERIALS AND METHODS

### Animals

Adult male Sprague-Dawley rats (Charles River Laboratories; 250–500g at the time of experiments) were obtained and allowed at least one week acclimation to housing facilities. Rats were singly housed at least 5 days before behavioral experiments or tissue collection and maintained on a 12h light/dark cycle, with ad libitum access to food and water. Animal use and procedures were in accordance with NIH guidelines and approved by the Yale University Animal Care and Use Committee.

### Experimental Design

Experiments 1: To examine the effects of systemic opioid receptor blockade on ketamine’s behavioral actions, rats received naltrexone (Sigma-Aldrich #N3136, St. Louis, Missouri; 20 mg/kg, i.p.) or saline injection 30 minutes prior to ketamine (Sigma-Aldrich #K2753; 10 mg/kg, i.p.) or saline treatment. Naltrexone at this dose has been frequently used in preclinical studies [20–22], although it may lose some of its selectivity for the μ-OR and also bind to δ-, and κ-ORs at this dose [23–26]. Behavioral testing was carried out starting 24 hours after ketamine treatment.
Experiment 2: To examine the effects of local opioid receptor blockade in the mPFC on ketamine’s behavioral actions, mPFC-cannulated rats were bilaterally infused with naltrexone (20 μg/0.5 μl/side) 30 minutes prior to ketamine (10 mg/kg, i.p.) or saline treatment. This naltrexone dose was chosen based on previous studies demonstrating the effects of intracranially microinjected naltrexone [27, 28]. Behavioral testing was carried out starting 24 hours after ketamine treatment.
Experiment 3: ELISA and qPCR were performed with mPFC and hypothalamus collected 1 or 24 hours following ketamine (10 mg/kg, i.p.) or saline treatment to examine ketamine’s effects on the presence of β-endorphin and expression of opioid receptors in the mPFC and on the expression of β-endorphin precursor, proopiomelanocortin (POMC), in the hypothalamus.
Experiment 4: To examine the effects of local neutralization of β-endorphin on ketamine’s behavioral, mPFC-cannulated rats were bilaterally infused with anti-β-endorphin antibody (0.5 μg/0.5 μl/side; Phoenix Pharmaceuticals #G-022–33, Burlingame, California) or control IgG 30 minutes prior to ketamine (10 mg/kg, i.p.) or saline treatment. Behavioral testing was carried out starting 24 hours after ketamine treatment.
Experiment 5: To examine the effects of systemic opioid receptor blockade and local neutralization of β-endorphin on ketamine’s molecular actions, rats received treatments as described in Experiments 1 and 4, and mPFC was collected 1 hour or 24 hours after ketamine treatment for Western blot analysis.

### mPFC Cannulation and Infusion Procedures

Rats were anaesthetized with a mixture of ketamine (75 mg/kg) and xylazine (5 mg/kg), and bilateral guide cannulae (P1 Technologies Inc., Roanoke, Virginia: 22-gauge) were implanted 0.5 mm above the infusion site at the following coordinates: from bregma: +3.0 mm anterior/posterior; +/− 1.0 mm medial/lateral; and − 4.0 mm dorsal/ventral. Following 9–14 days of recovery, rats were bilaterally infused with naltrexone or an anti-β-endorphin antibody at a rate of 0.2 μl/min, using a microinfusion pump (Harvard Apparatus, Holliston, Massachusetts). The needle was left in place for 2.5 minutes after the injection to allow complete dispersion of the solution. Naltrexone was dissolved in saline; control rats were infused with 0.9% saline. The anti-β-endorphin antibody was reconstituted following manufacturer’s instructions; rabbit control puri ed IgG (Phoenix Pharmaceuticals #NRG-500) was used as a control.

### Forced Swim Test (FST)

FST was conducted as previously described [29]. Rats were exposed to a 15-minute pre-swim in 25 ± 1°C water in a Plexiglas cylinder (65 cm height, 30 cm diameter). 24 hours following the pre-swim, rats were treated as described above and then placed in the swim tank for 10 minutes. Data were analyzed by scoring immobility time during minutes 2–6.

### Female Urine Sning Test (FUST)

FUST was conducted as previously described [29]. Rats were habituated to a sterile cotton-tipped applicator placed into their home cage for 1 hour, and then exposed to a water-dipped cotton-tipped applicator for 5 minutes. After a 45-minute interval, rats were exposed to a cotton-tipped applicator infused with 75 μl fresh urine from females of the same strain for 5 minutes, during which the time spent sniffing the cotton-tipped applicator was measured. Time spent biting the cotton-tip was excluded from analysis.

### Novelty Suppressed Feeding Test (NSFT)

NSFT was conducted as previously described [29]. Rats were food deprived for at least 20 hours and then placed in an open field with one food pellet in the center. The latency to feed was measured, with a cut-off time of 15 minutes. After NSFT, home cage feeding during a 15-minute period was measured to verify motivation to feed.

### Locomotor Activity

Rats were placed in testing cages (46 cm × 23 cm × 20 cm) for 30 minutes, during which the number of beam breaks was measured using Med-PC software (Med Associates, Fairfax, Vermont).

### Protein and RNA sample preparation, Western Blot, and qPCR analysis

Crude synaptosomal fraction or total homogenate of rat mPFC were prepared and analyzed by Western blot. RNA from mPFC or hypothalamus was extracted by RNeasy Mini Kit (Qiagen #74104, Hilden, Germany), reverse transcribed and subjected to qPCR, as detailed in Supplemental Material.

#### Primary hypothalamic culture and in vitro ketamine treatment

Primary hypothalamic culture prepared from E18 embryos was treated with 0.5 μM ketamine on day 12 *in vitro*, as detailed in Supplemental Material.

### β-endorphin ELISA analysis

Rat mPFC was collected 1 hour or 24 hours following ketamine injection and homogenized in PBS. β-endorphin levels were measured using QuickDetect^™^ beta-Endorphin (Rat) ELISA Kit (BioVision, Milpitas, California #E4460) according to the manufacturer’s instructions. Protein concentrations in each sample were measured using a Pierce BCA Protein Assay Kit (Thermo Scientific, Waltham, Massachusetts) and results are presented as pg of β-endorphin in 1 mg of protein in the sample.

### Statistics

Statistical analyses were performed using GraphPad Prism (San Diego, California). Values were excluded only if they were detected as outliers by Grubb’s test. Comparisons between two groups were made using Student’s t test. Correlation was calculated using Pearson’s r. Comparisons for four groups were made using two-way analysis of variance (ANOVA) followed by Sidak’s multiple comparisons, as indicated in the Figure Legends and Supplemental Material (Table S1). All tests are two-sided. All data are presented as mean ± s.e.m..

## RESULTS

### Systemic naltrexone pretreatment blocks the antidepressant-like actions of ketamine

**Figure 1:**
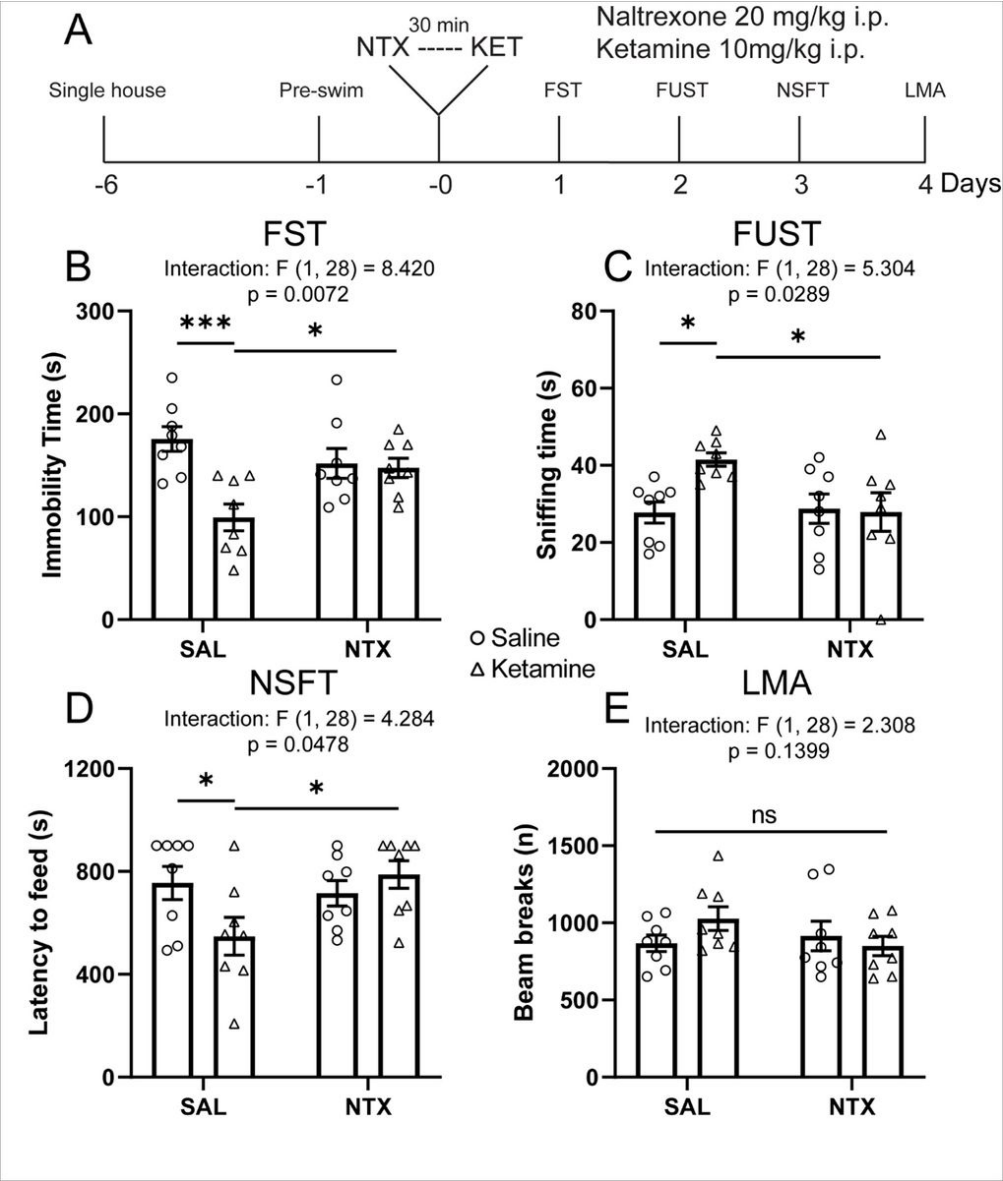
Systemic naltrexone pretreatment blocks the antidepressant-like actions of ketamine **(A)** Schematic timeline of systemic naltrexone pretreatment, ketamine treatment, and behavioral testing. Systemic naltrexone pretreatment blocks (**B**) the reduction in immobility time in the FST (ketamine × naltrexone interaction F_(1, 28)_ = 8.420, p = 0.0072; see Table S1 for full statistical results), (**C**) the increase in sniffing time in the FUST (interaction F_(1, 28)_ = 5.304, p = 0.0289), and (**D**) the reduction of latency to feed in the NSFT induced by ketamine (interaction F_(1, 28)_ = 4.284, p = 0.0478), without affecting locomotor activity (interaction F_(1, 28)_ = 2.308, p = 0.1399) (**E**). Two-way ANOVA followed by Sidak’s post hoc test. n = 8/group. Post hoc significant effects indicated as: * p<0.05, *** p<0.001. ns, nonsigni cant; SAL, saline; NTX, naltrexone; FST, forced swim test; FUST, female urine sniffing test; NSFT, novelty suppressed feeding test; LMA, locomotion.

To investigate whether the endogenous opioid system is required for the antidepressant-like actions of ketamine, we treated rats with naltrexone (20 mg/kg, i.p.) 30 minutes before ketamine (10 mg/kg, i.p.; Fig. 1A). Rats were tested in a series of behavioral paradigms, including forced swim test (FST), female urine sniffing test (FUST), novelty suppressed feeding test (NSFT), and locomotor activity (LMA), starting 24 hours after ketamine treatment. In the FST, a model of behavioral despair, ketamine significantly reduced immobility time in saline-pretreated rats; this effect was completely blocked by naltrexone pretreatment (Fig. 1B). In FUST, a model in which less time spent sniffing female urine indicates anhedonia in males, ketamine significantly increased female urine sniffing time in saline- but not naltrexone-pretreated rats (Fig. 1C). In the NSFT, a paradigm in which longer latency to feed is considered an anxiety-like behavior, ketamine significantly shortened latency to feed in saline- but not naltrexonepretreated rats (Fig. 1D), without changing home cage food consumption (Figure S1). Locomotor activity was not affected by either naltrexone pretreatment or ketamine treatment (Fig. 1E). Together, these results suggest that activation of opioid receptors is required for the behavioral effects of ketamine.

### Intra-mPFC naltrexone infusion blocks the antidepressantlike actions of ketamine

**Figure 2:**
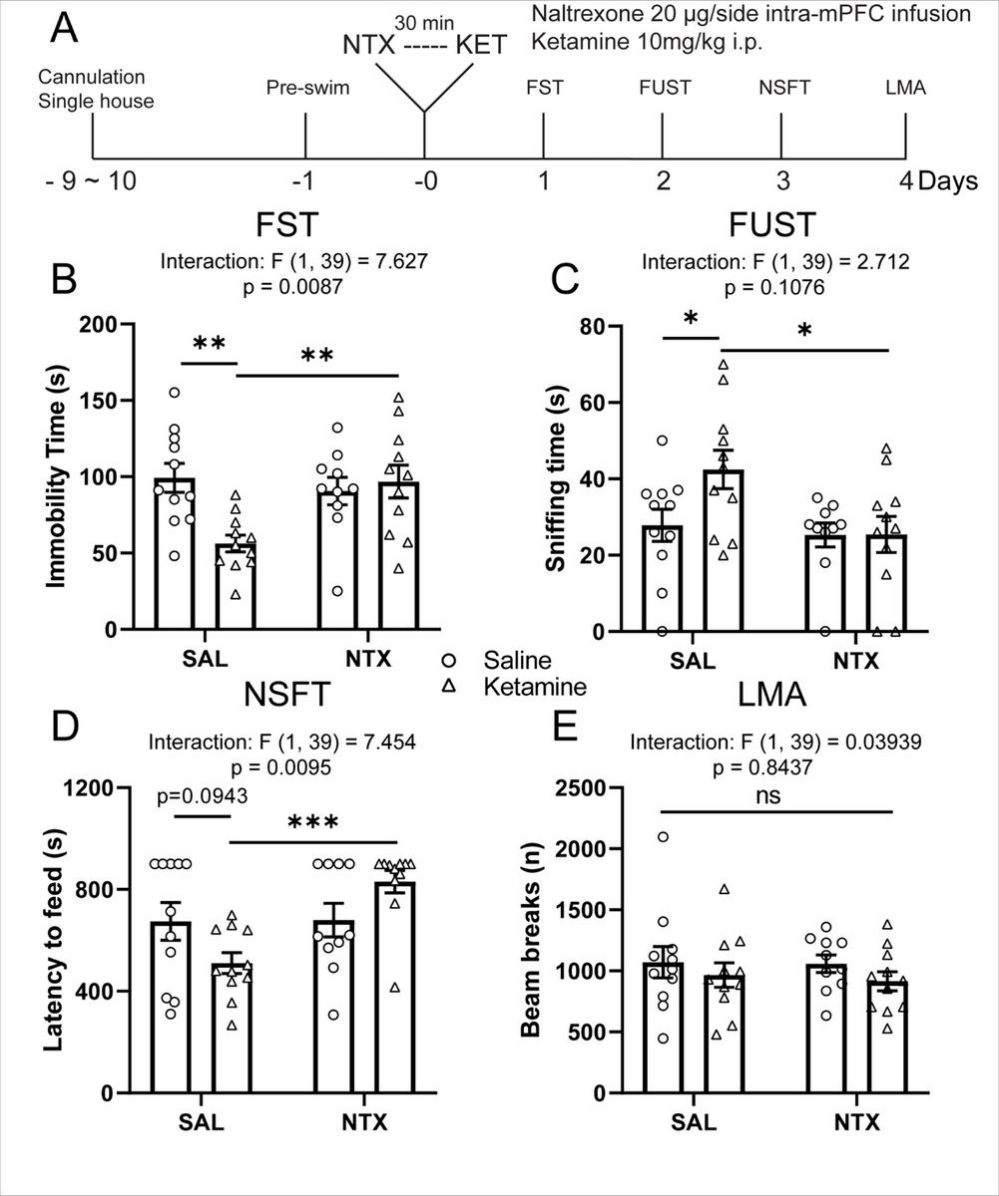
Intra-mPFC naltrexone infusion blocks the antidepressant-like actions of ketamine. **(A)** Schematic timeline of intra-mPFC naltrexone pretreatment, ketamine treatment and behavioral testing. Intra-mPFC naltrexone pretreatment blocks (**B**) the reduction in immobility time in the FST (ketamine × naltrexone interaction: F_(1, 39)_ = 7.627, p = 0.0087), (**C**) the increase in sniffing time in the FUST (naltrexone main effect: F_(1, 39)_ = 4.927; p = 0.0323), and (**D**) the reduction of latency to feed in the NSFT induced by ketamine (interaction: F_(1, 39)_ = 7.454, p = 0.0095), without affecting locomotor activity (interaction: F_(1, 39)_ = 0.03939, p = 0.8437) (**E**). Two-way ANOVA followed by Sidak’s post hoc test. n = 11/group for SAL/SAL, SAL/KET, and NTX/KET; n = 10 for NTX/SAL. Post hoc significant effects indicated as: * p<0.05, ** p<0.01, *** p<0.001. ns, nonsignificant; SAL, saline; NTX, naltrexone; FST, forced swim test; FUST, female urine sniffing test; NSFT, novelty suppressed feeding test; LMA, locomotion.

As the mPFC plays an important role in regulating ketamine’s antidepressant-like effects [11, 12], we sought to determine whether localized blockade of ORs using targeted naltrexone infusion into the mPFC would be sufficient to attenuate the behavioral effects of ketamine. We infused naltrexone (20 μg/0.5 μl/side) or saline into the mPFC of cannulated rats 30 minutes before ketamine treatment (10 mg/kg, i.p.) and subjected the rats to behavioral tests starting 24 hours later (Fig. 2A). In saline-pretreated rats, ketamine induced significant behavioral effects in FST (Fig. 2B), FUST (Fig. 2C), and NSFT (Fig. 2D); all effects were absent in naltrexone-pretreated rats. There were no effects of ketamine, with or without naltrexone, on home cage feeding (Figure S2) or locomotor activity (Fig. 2E).

### Ketamine induces β-endorphin release in mPFC

While naltrexone is a nonselective OR antagonist, it has the highest affinity for μ-ORs [30, 31]. β-endorphin is the primary endogenous agonist for μ-ORs, and its presence in the cortex has been documented [32].

**Figure 3:**
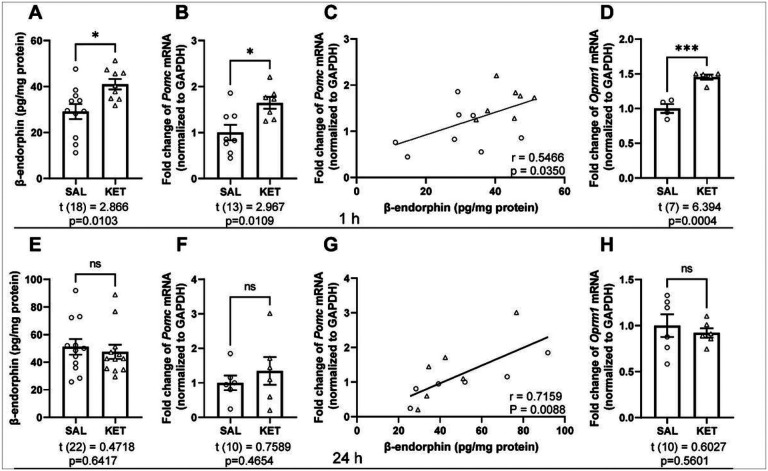
Ketamine increases endogenous opioid activity in the mPFC and hypothalamus. **(A)** Ketamine treatment (10 mg/kg, i.p.) significantly increases β-endorphin levels in the mPFC (t_(18)_ = 2.866, p = 0.0103; n = 11 for SAL, n = 9 for KET) and (**B**) *Pomc* mRNA expression in the hypothalamus (t_(13)_ = 2.967, p = 0.0109; n = 8 for SAL, n = 7 for KET) at 1h. (**C**) Elevated β-endorphin levels in the mPFC is positively correlated with increased Pomc mRNA levels in the hypothalamus (r = 0.5466, p = 0.0350). (**D**) Ketamine treatment significantly increases *Oprm1* mRNA expression in the mPFC at 1h (t_(7)_ = 6.394, p = 0.0004; n = 4 for SAL, n = 5 for KET). (**E**) At 24h, there is no change in β-endorphin levels in the mPFC (t_(22)_ = 0.4718, p = 0.6417; n = 12/group) and (**F**) there is no significant increase in *Pomc* mRNA in the hypothalamus (t_(10)_ = 0.7589, p = 0.4654; n = 6/group); (**G**) but its significant correlation with mPFC β-endorphin levels remains (r = 0.7159, p = 0.0088). (**H**) Ketamine treatment does not change *Oprm1* mRNA expression in the mPFC at 24h (t_(10)_ = 0.6027, p = 0.5601; n = 6/group). Student’s t test for A, B, D, E, F, H. Pearson’s r for C, G. * p<0.05, ** p<0.01, *** p<0.001. ns, nonsignificant; SAL, saline; KET, ketamine.

Ketamine significantly increased β-endorphin levels in the mPFC, measured using ELISA in dissected tissue collected 1 hour after treatment (Fig. 3A). β-endorphin is derived from a precursor protein, proopiomelanocortin (POMC), encoded by the *Pomc* gene, which is primarily expressed in the arcuate nucleus (ARC) of hypothalamus, as well as in the pituitary gland [33]. To investigate whether ketamine concurrently influences *Pomc* expression in the hypothalamus, we measured *Pomc* mRNA using qPCR in tissue collected 1 hour following ketamine treatment. Ketamine significantly increased hypothalamic *Pomc* mRNA relative to saline-injected controls (Fig. 3B); change in *Pomc* was positively correlated with the increased β-endorphin level in the mPFC (Fig. 3C). In further support of this finding, increased β-endorphin, measured by ELISA, was released into the supernatant after ketamine treatment in hypothalamic neuronal cultures, at trend level (Figure S3A, B). Ketamine also significantly increased mPFC *Oprm1* mRNA (Fig. 3D), but not *Oprk1* mRNA (Figure S3C), at 1 hour.

At 24 hours, the elevation in β-endorphin levels in the mPFC was completely absent in the ketamine group (Fig. 3E), and there was no change in *Pomc* mRNA expression in the hypothalamus (Fig. 3F); however, hypothalamic *Pomc* expression remained positively correlated with β-endorphin level in the mPFC (Fig. 3G). Ketamine treatment did not significantly change the mRNA expression of *Oprm1* (Fig. 3H) and *Oprk1* (Figure S3D) in the mPFC at 24 hours. Thus, ketamine induced a rapid, non-sustained increase in β-endorphin and the expression of the gene that encodes its primary receptor, μ-OR, in the mPFC, and of the expression of the gene that encodes the β-endorphin precursor, POMC, in the hypothalamus.

### Intra-mPFC anti-β-endorphin antibody infusion blocks the antidepressant-like actions of ketamine

**Figure 4:**
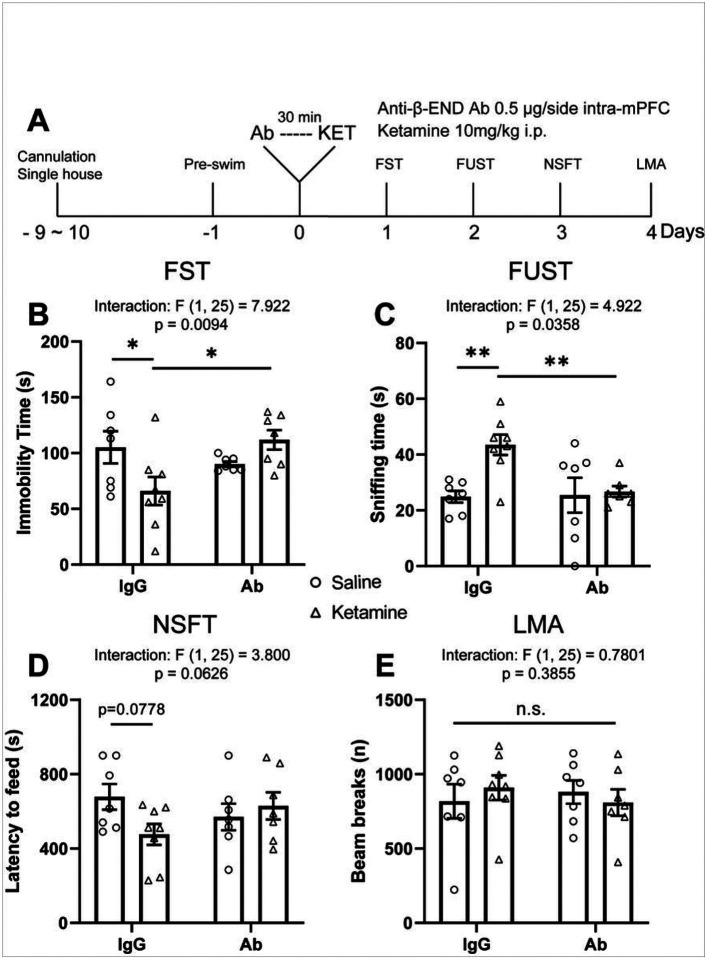
β-endorphin neutralization in the mPFC blocks the antidepressant-like actions and molecular effects of ketamine. (**A**) Schematic timeline of intra-mPFC anti-β-endorphin neutralizing antibody pretreatment, ketamine treatment, and behavioral testing. Intra-mPFC anti-β-endorphin neutralizing antibody pretreatment blocks (**B**) the reduction in immobility time in the FST (ketamine × antibody interaction: F_(1, 25)_ = 7.922, p = 0.0094), (**C**) the increase in sniffing time in the FUST (interaction: F_(1,25)_ = 4.922, p = 0.0358), (**D**) the trend-level reduction of latency to feed in the NSFT induced by ketamine (interaction: F_(1, 25)_ = 3.800, p = 0.0626), without affecting locomotor activity (interaction: F_(1, 25)_ = 0.7801, p = 0.3855) (**E**). Two-way ANOVA followed by Sidak’s post hoc test. n = 7 for IgG/SAL, n = 8 for IgG/KET, n = 7 for Ab/SAL, n = 7 for Ab/KET. Post hoc significant effects indicated as: * p<0.05, ** p<0.01. ns, nonsignificant; IgG, control IgG; Ab, anti-β-endorphin neutralizing antibody; FST, forced swim test; FUST, female urine sniffing test; NSFT, novelty suppressed feeding test; LMA, locomotion.

We next tested whether β-endorphin in the mPFC is necessary for the behavioral effects of ketamine. We infused rats with a β-endorphin neutralizing antibody (0.5 μg/0.5 μl/side) or control IgG 30 minutes before ketamine treatment (10 mg/kg, i.p.) and subjected them to behavioral tests starting 24 hours later (Fig. 4A). In control IgG-infused rats, ketamine produced robust behavioral effects, all of which were absent in the rats that received β-endorphin antibody infusion: FST (Fig. 4B), FUST (Fig. 4C) and NSFT (Fig. 4D). There was no effect on locomotor activity or home cage feeding (Fig. 4E and Figure S4). Intra-mPFC β-endorphin antibody infusion alone did not influence these behaviors.

### Systemic naltrexone pretreatment abolishes ketamine-induced molecular changes in the mPFC

**Figure 5:**
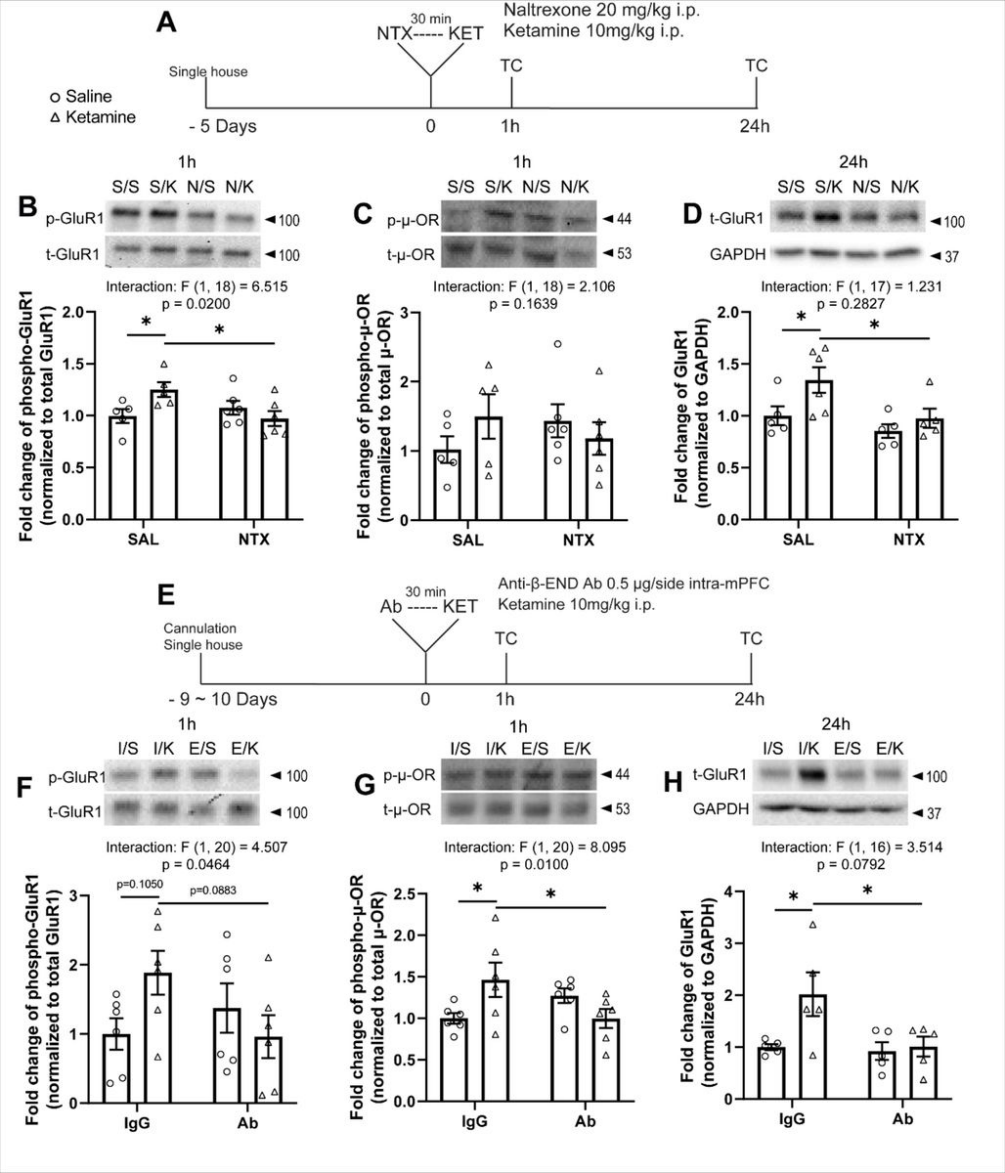
Systemic naltrexone pretreatment blocks the molecular effects of ketamine. **(A)** Schematic timeline of systemic naltrexone pretreatment, ketamine treatment, and tissue collection. Systemic naltrexone pretreatment blocks (**B**) the increase in phosphorylation of GluR1 (interaction: F_(1, 18)_ = 6.515, p = 0.0200) and (**C**) the trend-level increase in phosphorylation of μ-ORs at Ser375 in the total homogenate induced by ketamine at 1h. (**D**) Systemic naltrexone pretreatment blocks total GluR1 levels in the synaptosomes induced by ketamine at 24h (ketamine main effect: F_(1, 17)_ = 5.492, p = 0.0315; naltrexone main effect: F_(1, 17)_ = 6.673, p = 0.0193). (**E**) Schematic timeline of intra-mPFC anti-β-endorphin neutralizing antibody pretreatment, ketamine treatment, and tissue collection. Intra-mPFC anti-β-endorphin neutralizing antibody pretreatment blocks (**F**) the trend-level increase in GluR1 phosphorylation (interaction: F_(1, 20)_ = 4.507, p = 0.0464) and (**G**) the increase in phosphorylation of μ-ORs at Ser375 (interaction: F_(1, 20)_ = 8.095, p = 0.0100) in the total homogenate at 1h. (**H**) At 24h, intra-mPFC anti-β-endorphin neutralizing antibody pretreatment blocks the increase in total GluR1 levels in the synaptosomes at 24h (interaction: F_(1, 16)_ = 3.514, p = 0.0792; ketamine main effect: F_(1, 16)_ = 4.980, p = 0.0403; antibody main effect: F_(1, 16)_ = 4.809, p = 0.0434). Two-way ANOVA followed by Sidak’s post hoc test. n = 5 for S/S and S/K, n = 6 for N/S and N/K in B and C; n = 5 for S/S, N/S, N/K and n = 6 for S/K in D; n = 6/group in F and G; n = 5/group in H. Post hoc significant effects indicated as: * p<0.05. SAL/S, saline; NTX/N, naltrexone; K, ketamine; IgG/I, control IgG; Ab/E, anti-β-endorphin neutralizing antibody.

To determine the molecular mechanisms underlying impaired ketamine response, we examined the effect of systemic naltrexone pretreatment on ketamine-induced phosphorylation of AMPA receptor subunit GluR1, which has been implicated in ketamine’s antidepressant-like actions [2, 34, 35], using Western blotting. mPFC tissue was collected 1 hour after ketamine injection, preceded by naltrexone or saline (Fig. 5A). Ketamine significantly increased phosphorylation of GluR1 in the total homogenate (Fig. 5B); this was blocked by naltrexone pretreatment. Similarly, ketamine induced numerically increased phosphorylation of μ-OR, which was partially blocked by naltrexone pretreatment at 1 hour, though these effects did not reach statistical significance (Fig. 5C).

We next determined the effect of naltrexone pretreatment on the ketamine-induced elevation in new synthesis of GluR1 in the mPFC [12, 36]. 24 hours after ketamine treatment, GluR1 protein levels were significantly increased in mPFC synaptosomes in saline-pretreated rats, while no significant changes were observed in naltrexone-pretreated group (Fig. 5D). These results indicate that opioid receptor activation is required for synaptic changes induced by ketamine in the mPFC.

### Intra-mPFC anti-β-endorphin antibody infusion blocks the molecular effects of ketamine

To determine whether β-endorphin in the mPFC is required for these molecular effects, we infused rats with a β-endorphin neutralizing antibody 30 minutes before ketamine treatment and collected mPFC 1 hour and 24 hours after ketamine (Fig. 5E). Increased phosphorylation of GluR1 (Fig. 5F) and μ-OR (Fig. 5G) in the total homogenate at 1 hour were both absent in rats pretreated with β-endorphin antibody. Increased GluR1 levels in synaptosomes at 24 hours (Fig. 5H) was similarly abolished by β-endorphin antibody pretreatment.

## DISCUSSION

We demonstrate that blockade of opioid receptors by a single dose of systemic naltrexone 30 minutes prior to ketamine treatment abolishes the effects of ketamine on behavioral despair, anhedonia-like, and anxiety-like behaviors in rats. This is consistent with several previous clinical and preclinical reports [13, 14, 16–18], though not with others [15, 19]. These discrepancies in the literature could be explained by the differences in the routes and doses of naltrexone administered, the timepoints at which naltrexone is administered, comorbidity of alcohol use disorder with depression in one clinical study [15], and prior exposure to stress, which influences expression of opioid receptor [37], in one preclinical study [19]. Additional preclinical studies employing stress exposure, comprehensive behavioral phenotyping, and various doses of naltrexone are needed to clarify these discrepancies.

Intra-mPFC infusion of naltrexone prior to ketamine treatment also blocks the behavioral effects of ketamine, and systemic naltrexone pretreatment blocks ketamine-induced molecular changes in the mPFC. These results indicate that opioid signaling in the mPFC plays a central role in regulating ketamine’s actions. This importantly extends recent research on the interplay between ketamine and the opioid system by pinpointing a critical site of action, although our current data do not exclude contributions from other locations.

Naltrexone can bind to μ-ORs, κ-ORs, and δ-ORs at the dose we used [23–26], and β-endorphin binds to both μ-ORs and δ-ORs, with a lower affinity for κ-ORs. Recent studies have reported that pharmacological blockade of κ-ORs abolished the behavioral effects of repeated ketamine administration in the FST in mice [17], and that δ-OR agonists produce antidepressant-like effects [38]. We cannot rule out the contribution of κ-ORs and δ-ORs in mediating ketamine’s actions. Future studies using more specific antagonists for μ-ORs, κ-ORs, and δ-ORs and more comprehensive behavioral testing and molecular characterization will be needed to unambiguously determine the specific type(s) of ORs that mediate ketamine’s actions. However, phosphorylation of μ-ORs and upregulation of POMC are suggestive of a μ-OR mediated mechanism; this remains our leading hypothesis to explain these effects.

*Oprm1* is expressed in GABAergic and glutamatergic neurons and on astrocytes in the cortex [39, 40]. Recent studies suggest that ketamine blocks NMDARs on GABAergic interneurons, resulting in disinhibition and a burst of glutamate that produces synaptic and behavioral effects [41–43]. Our results support an intriguing model that merits further study: ketamine induces release of β-endorphin into the mPFC, which activates μ-ORs on GABAergic interneurons and/or astrocytes. Inhibition of interneurons and release of glutamate from astrocytes, both of which are known effect of μ-OR activation [44–47], could synergize with the direct effects of ketamine on GABAergic interneurons and consequent disinhibition of pyramidal neurons and surge in synaptic glutamate [41–43], thus contributing to the initiation of rapid and sustained antidepressant-like effects. However, alternative interpretations exist. Ketamine may directly activate ORs: it has an appreciable binding affinity for both μ-ORs (K_i_ = 42.1 μM) and κ-ORs (K_i_ = 28.1 μM) [3]. However, even if ketamine does meaningfully engage ORs during single-dose treatment, since ketamine has a higher affinity for κ-ORs than for μ-ORs, and κ-OR agonists have dysphoric and aversive properties [48], one would expect direct agonist effects to be biased towards the pro-depressant effects produced by κ-OR activation. This is not what we observe.

We find evidence for stimulation of β-endorphin release by ketamine in hypothalamic neuronal culture (Figure S3A, B), as has previously been reported in pituitary cell culture [49]. Previous studies suggest that ketamine induces endogenous opioid release *in vivo* [50, 51]. We document elevated β-endorphin levels in the mPFC, and a correlated increase in Pomc mRNA levels in the hypothalamus, 1 hour following ketamine treatment *in vivo*; both return to baseline at 24 hours but remain correlated with one another. These observations suggest that ketamine activates ARC POMC neurons, directly or indirectly, leading to rapid and transient increase in β-endorphin in the mPFC, which collectively with other processes [41–43], initiates the antidepressant-like effects. ARC POMC neurons send projections to cortical areas and limbic system [52], so it is possible that β-endorphin is directly released by the ARC POMC neuron terminals in the mPFC. Alternatively, β-endorphin released in the hypothalamus may be transported via cerebrospinal fluid in the ventricular system to the mPFC by volume transmission [53].

β-endorphin has been implicated in the pathophysiology and treatment of depression [7, 8]. Plasma β-endorphin levels are correlated with certain clinical symptoms of depression [54] and are increased by several antidepressant treatments [55, 56]. Release of endogenous opioid(s) targeting μ-ORs in the PFC has been observed after high-intensity exercise [57], which has been linked to improved mood [58]. Fluoxetine, a selective serotonin reupdate inhibitor, has been demonstrated to induce β-endorphin release in the ARC and nucleus accumbens [59]. Although our observations that ketamine increases β-endorphin levels in the mPFC *in vivo* and that intra-mPFC pretreatment with anti-β-endorphin neutralizing antibody blocks the antidepressant-like effects of ketamine suggest a causal relationship between β-endorphin in the mPFC and ketamine’s actions, we did not directly monitor changes in the extracellular level of β-endorphin in the mPFC in response to ketamine (the increase shown in Fig. 3A was in total mPFC tissue), nor did we examine potential ketamine-induced β-endorphin release in other brain regions. Future study using *in vivo* microdialysis could better characterize the temporal profile of ketamine-induced β-endorphin release in the mPFC. Future examination of multiple brain regions might also identify other regions where β-endorphin may have a role in mediating ketamine’s actions.

Previous studies have implicated brain-derived neurotrophic factor (BDNF) and vascular endothelial growth factor (VEGF) in the antidepressant-like actions of ketamine [60, 61]. Whether these trophic factors and their respective signaling act in parallel with or subsequently to β-endorphin remains to be determined. β-endorphin has been reported to increase BDNF expression in the PFC and hippocampus [62], and μ-OR agonists activate VEGF receptors [63], suggesting the possibility that BDNF and VEGF signaling could be downstream of β-endorphin. However, it is also possible that ketamine induces release of β-endorphin, BDNF, and VEGF independently and they then act interdependently, together with other processes, to mediate ketamine’s antidepressant-like effects.

It has been shown that ketamine and other agents with rapid antidepressant-like properties rapidly induces GluR1 phosphorylation in multiple brain regions, including mPFC [2, 34–36, 64], and that GluR1 phosphorylation is required for the rapid and sustained antidepressant-like effects of ketamine and subsequent increase in synaptic GluR1 levels [35]. Our results indicate that activation of opioid receptors and presence of β-endorphin in the mPFC are required for ketamine-induced increase in GluR1 phosphorylation and elevated synaptosomal GluR1 levels in the mPFC. μ-OR agonists have been shown to increase protein kinase A and calcium/calmodulin-dependent protein kinase II activity *in vivo* [65, 66], which can in turn phosphorylates GluR1 [67, 68], mediating its role in regulating synaptic delivery, and incorporation of GluR1-containing AMPA receptors into synapses [69]. β-endorphin leads to phosphorylation of μ-ORs at Ser375 [70].

Preclinical studies have begun to reveal sex differences in response to ketamine. Females are sensitive to lower dose of ketamine and exhibit stronger behavioral response in some contexts [77]. Sex differences have also been reported in β-endorphin levels in multiple brain regions, both at baseline and under various experimental conditions [78, 79]. Because of these reported effects of sex, we focused here on male rats, to reduce the number of variables at play. It will be important to examine potential sexual dimorphisms in the reported effects in future studies.

Our data suggest that β-endorphin in the mPFC can contribute to antidepressant-like effects. Previous studies have provided conflicting evidence on this question. In mice, morphine reduces immobility time in the FST and tail suspension test (TST) [80, 81]. In rats, however, morphine does not influence the immobility time in the FST [16, 82]. Early clinical studies documented antidepressant effects induced by intravenous β-endorphin infusion [83–85]. Within central nervous system, intracerebroventricular infusion of β-endorphin increases *Bdnf* mRNA expression in the PFC and hippocampus [62]; this is similar to the effects seen following chronic conventional antidepressant treatments [86] and acute ketamine administration [87]. Interestingly, one recent study found that endogenous and exogenous opioids act on GABAergic and glutamatergic neurons, respectively, to mediate behavioral effects [88]. Therefore, the lack of consistent effects from exogenous μ-OR agonists cannot rule out the possibility that endogenous β-endorphin possesses rapid antidepressant potential.

In summary, our study demonstrates that β-endorphin and opioid receptor activation in the mPFC are required for the behavioral and molecular actions of ketamine in a well-established rat model. These findings are consistent with accumulating evidence implicating endogenous opioid signaling in the rapid antidepressant effects of ketamine. Importantly, our results suggest a potential mechanism by which ketamine produces antidepressant-like actions: by increasing β-endorphin release, which in turn activates μ-ORs in the mPFC. This work lays the foundation for future studies to further delineate these mechanisms to inform the development of next-generation rapidly acting antidepressant agents.

## References

[1] Berman RM, Cappiello A, Anand A, Oren DA, Heninger GR, Charney DS, Antidepressant effects of ketamine in depressed patients. Biol Psychiatry. 2000;47(4):351–4. doi:10.1016/s0006-3223(99)00230-9 10686270

[2] Maeng S, Zarate CA Jr., Du J, Schloesser RJ, McCammon J, Chen G, Cellular mechanis msunderlying the antidepressant effects of ketamine: role of alpha-amino-3-hydroxy-5-methylisoxazole-4-propionic acid receptors. Biol Psychiatry. 2008;63(4):349–52. doi:10.1016/j.biopsych.2007.05.028 17643398

[3] Hirota K, Okawa H, Appadu BL, Grandy DK, Devi LA, Lambert DG. Stereoselective interaction of ketamine with recombinant mu, kappa, and delta opioid receptors expressed in Chinese hamster ovary cells. Anesthesiology. 1999;90(1):174–82. doi:10.1097/00000542-199901000-00023 9915326

[4] Drolet G, Dumont EC, Gosselin I, Kinkead R, Laforest S, Trottier JF. Role of endogenous opioid system in the regulation of the stress response. Progress in neuro-psychopharmacology & biological psychiatry. 2001;25(4):729–41. doi:10.1016/s0278-5846(01)00161-0 11383975

[5] Millan MJ. Multiple opioid systems and pain. Pain. 1986;27(3):303–47. doi:10.1016/0304-3959(86)90158-2 3027643

[6] Trigo JM, Martin-Garcia E, Berrendero F, Robledo P, Maldonado R. The endogenous opioid system: a common substrate in drug addiction. Drug Alcohol Depend. 2010;108(3):183–94. doi:10.1016/j.drugalcdep.2009.10.011 19945803

[7] Pecina M, Karp JF, Mathew S, Todtenkopf MS, Ehrich EW, Zubieta JK. Endogenous opioid system dysregulation in depression: implications for new therapeutic approaches. Molecular psychiatry. 2019;24(4):576–87. doi:10.1038/s41380-018-0117-2 29955162 PMC6310672

[8] Hegadoren KM, O’Donnell T, Lanius R, Coupland NJ, Lacaze-Masmonteil N. The role of beta-endorphin in the pathophysiology of major depression. Neuropeptides. 2009;43(5):341–53. doi:10.1016/j.npep.2009.06.004 19647870

[9] Hsu DT, Sanford BJ, Meyers KK, Love TM, Hazlett KE, Walker SJ, It still hurts: altered endogenous opioid activity in the brain during social rejection and acceptance in major depressive disorder. Molecular psychiatry. 2015;20(2):193–200. doi:10.1038/mp.2014.185 25600108 PMC4469367

[10] Lutz PE, Almeida D, Filliol D, Jollant F, Kieffer BL, Turecki G. Increased functional coupling of the mu opioid receptor in the anterior insula of depressed individuals. Neuropsychopharmacology. 2021;46(5):920–7. doi:10.1038/s41386-021-00974-y 33531622 PMC8115105

[11] Duman RS, Aghajanian GK, Sanacora G, Krystal JH. Synaptic plasticity and depression: new insights from stress and rapid-acting antidepressants. Nature medicine. 2016;22(3):238–49. doi:10.1038/nm.4050 PMC5405628 26937618

[12] Li N, Lee B, Liu RJ, Banasr M, Dwyer JM, Iwata M, mTOR-dependent synapse formation underlies the rapid antidepressant effects of NMDA antagonists. Science. 2010;329(5994):959–64. doi:10.1126/science.1190287 20724638 PMC3116441

[13] Williams NR, Heifets BD, Bentzley BS, Blasey C, Sudheimer KD, Hawkins J, Attenuation of antidepressant and antisuicidal effects of ketamine by opioid receptor antagonism. Molecular psychiatry. 2019;24(12):1779–86. doi:10.1038/s41380-019-0503-4 31467392

[14] Williams NR, Heifets BD, Blasey C, Sudheimer K, Pannu J, Pankow H, Attenuation of Antidepressant Effects of Ketamine by Opioid Receptor Antagonism. The American journal of psychiatry. 2018;175(12):1205–15. doi:10.1176/appi.ajp.2018.18020138 30153752 PMC6395554

[15] Yoon G, Petrakis IL, Krystal JH. Association of Combined Naltrexone and Ketamine With Depressive Symptoms in a Case series of Patients With Depression and Alcohol Use Disorder. JAMA Psychiatry. 2019;76(3):337–8. doi:10.1001/jamapsychiatry.2018.3990 30624551 PMC6439824

[16] Klein ME, Chandra J, Sheriff S, Malinow R. Opioid system is necessary but not su cient for antidepressive actions of ketamine in rodents. Proceedings of the National Academy of Sciences of the United States of America. 2020;117(5):2656–62. doi:10.1073/pnas.1916570117 31941713 PMC7007545

[17] Wulf HA, Browne CA, Zarate CA, Lucki I. Mediation of the behavioral effects of ketamine and (2R,6R)-hydroxynorketamine in mice by kappa opioid receptors. Psychopharmacology. 2022;239(7):2309–16. doi:10.1007/s00213-022-06118-4 35459958

[18] Zhang F, Hillhouse TM, Anderson PM, Koppenhaver PO, Kegen TN, Manicka SG, Opioid receptor system contributes to the acute and sustained antidepressant-like effects, but not the hyperactivity motor effects of ketamine in mice. Pharmacology, biochemistry, and behavior. 2021;208:173228. doi:10.1016/j.pbb.2021.173228 34224734

[19] Zhang K, Hashimoto K. Lack of Opioid System in the Antidepressant Actions of Ketamine. Biol Psychiatry. 2019;85(6):e25–e7. doi:10.1016/j.biopsych.2018.11.006 30545521

[20] Le Merrer J, Stephens DN. Food-induced behavioral sensitization, its cross-sensitization to cocaine and morphine, pharmacological blockade, and effect on food intake. J Neurosci. 2006;26(27):7163–71. doi:10.1523/jneurosci.5345-05.2006 16822973 PMC6673946

[21] Rezvani AH, Overstreet DH, Vaidya AH, Zhao B, Levin ED. Carisbamate, a novel antiepileptic candidate compound, attenuates alcohol intake in alcohol-preferring rats. Alcoholism, clinical and experimental research. 2009;33(8):1366–73. doi:10.1111/j.1530-0277.2009.00966.x 19413647

[22] Ruegsegger GN, Brown JD, Kovarik MC, Miller DK, Booth FW. Mu-opioid receptor inhibition decreases voluntary wheel running in a dopamine-dependent manner in rats bred for high voluntary running. Neuroscience. 2016;339:525–37. doi:10.1016/j.neuroscience.2016.10.020 27743985

[23] Paterson SJ, Corbett AD, Gillan MG, Kosterlitz HW, McKnight AT, Robson LE. Radioligands for probing opioid receptors. J Recept Res. 1984;4(1–6):143–54. doi:10.3109/10799898409042545 6098650

[24] Takemori AE, Portoghese PS. Comparative antagonism by naltrexone and naloxone of mu, kappa, and delta agonists. European journal of pharmacology. 1984;104(1–2):101–4. doi:10.1016/0014-2999(84)90374-1 6094203

[25] Williams KL, Broadbridge CL. Potency of naltrexone to reduce ethanol self-administration in rats is greater for subcutaneous versus intraperitoneal injection. Alcohol. 2009;43(2):119–26. doi:10.1016/j.alcohol.2008.11.003 19251113 PMC2692629

[26] Childers SR, Creese I, Snowman AM, Synder SH. Opiate receptor binding affected differentially by opiates and opioid peptides. European journal of pharmacology. 1979;55(1):11–8. doi:10.1016/0014-2999(79)90142-0 220062

[27] MacDonald AF, Billington CJ, Levine AS. Effects of the opioid antagonist naltrexone on feeding induced by DAMGO in the ventral tegmental area and in the nucleus accumbens shell region in the rat. Am J Physiol Regul Integr Comp Physiol. 2003;285(5):R999–R1004. doi:10.1152/ajpregu.00271.2003 12907414

[28] Mitchell JM, Bergren LJ, Chen KS, Rowbotham MC, Fields HL. Naltrexone aversion and treatment efficacy are greatest in humans and rats that actively consume high levels of alcohol. Neurobiology of disease. 2009;33(1):72–80. doi:10.1016/j.nbd.2008.09.018 18955144

[29] Kato T, Pothula S, Liu RJ, Duman CH, Terwilliger R, Vlasuk GP, Sestrin modulator NV-5138 produces rapid antidepressant effects via direct mTORC1 activation. J Clin Invest. 2019;129(6):2542–54. doi:10.1172/jci126859 30990795 PMC6546461

[30] Niciu MJ, Arias AJ. Targeted opioid receptor antagonists in the treatment of alcohol use disorders. CNS Drugs. 2013;27(10):777–87. doi:10.1007/s40263-013-0096-4 23881605 PMC4600601

[31] Verebey K, Mule SJ. Naltrexone pharmacology, pharmacokinetics, and metabolism: current status. Am J Drug Alcohol Abuse. 1975;2(3–4):357–63. doi:10.3109/00952997509005661 1227297

[32] Bernstein HG, Henning H, Seliger N, Baumann B, Bogerts B. Remarkable beta-endorphinergic innervation of human cerebral cortex as revealed by immunohistochemistry. Neuroscience letters. 1996;215(1):33–6. doi:10.1016/s0304-3940(96)12939-6 8880747

[33] Toda C, Santoro A, Kim JD, Diano S. POMC Neurons: From Birth to Death. Annu Rev Physiol. 2017;79:209–36. doi:10.1146/annurev-physiol-022516-034110 28192062 PMC5669621

[34] Jiang C, Lin WJ, Sadahiro M, Labonte B, Menard C, Pfau ML, VGF function in depression and antidepressant efficacy. Molecular psychiatry. 2018;23(7):1632–42. doi:10.1038/mp.2017.233 29158577 PMC5962361

[35] Zhang K, Xu T, Yuan Z, Wei Z, Yamaki VN, Huang M, Essential roles of AMPA receptor GluA1 phosphorylation and presynaptic HCN channels in fast-acting antidepressant responses of ketamine. Sci Signal. 2016;9(458):ra123. doi:10.1126/scisignal.aai7884 27965425 PMC5564288

[36] Jiang C, Lin WJ, Labonte B, Tamminga CA, Turecki G, Nestler EJ, VGF and its C-terminal peptide TLQP-62 in ventromedial prefrontal cortex regulate depression-related behaviors and the response to ketamine. Neuropsychopharmacology. 2019;44(5):971–81. doi:10.1038/s41386-018-0277-4 30504797 PMC6462025

[37] Nikulina EM, Arrillaga-Romany I, Miczek KA, Hammer RP Jr., Long-lasting alteration in mesocorticolimbic structures after repeated social defeat stress in rats: time course of mu-opioid receptor mRNA and FosB/DeltaFosB immunoreactivity. The European journal of neuroscience. 2008;27(9):2272–84. doi:10.1111/j.1460-9568.2008.06176.x 18445218 PMC2442756

[38] Saitoh A, Yamada M. Antidepressant-like Effects of delta Opioid Receptor Agonists in Animal Models. Current neuropharmacology. 2012;10(3):231–8. doi:10.2174/157015912803217314 23449756 PMC3468877

[39] Maduna T, Audouard E, Dembele D, Mouzaoui N, Reiss D, Massotte D, Microglia Express Mu Opioid Receptor: Insights From Transcriptomics and Fluorescent Reporter Mice. Front Psychiatry. 2018;9:726. doi:10.3389/fpsyt.2018.00726 30662412 PMC6328486

[40] Yao Z, van Velthoven CTJ, Nguyen TN, Goldy J, Sedeno-Cortes AE, Baftizadeh F, A taxonomy of transcriptomic cell types across the isocortex and hippocampal formation. Cell. 2021;184(12):3222–41 e26. doi:10.1016/j.cell.2021.04.021 34004146 PMC8195859

[41] Moghaddam B, Adams B, Verma A, Daly D. Activation of glutamatergic neurotransmission by ketamine: a novel step in the pathway from NMDA receptor blockade to dopaminergic and cognitive disruptions associated with the prefrontal cortex. J Neurosci. 1997;17(8):2921–7. doi:10.1523/jneurosci.17-08-02921.1997 9092613 PMC6573099

[42] Widman AJ, McMahon LL. Disinhibition of CA1 pyramidal cells by low-dose ketamine and other antagonists with rapid antidepressant efficacy. Proceedings of the National Academy of Sciences of the United States of America. 2018;115(13):E3007–E16. doi:10.1073/pnas.1718883115 29531088 PMC5879689

[43] Gerhard DM, Pothula S, Liu RJ, Wu M, Li XY, Girgenti MJ, GABA interneurons are the cellular trigger for ketamine’s rapid antidepressant actions. J Clin Invest. 2020;130(3):1336–49. doi:10.1172/JCI130808 31743111 PMC7269589

[44] Milner TA, Drake CT. Ultrastructural evidence for presynaptic mu opioid receptor modulation of synaptic plasticity in NMDA-receptor-containing dendrites in the dentate gyrus. Brain research bulletin. 2001;54(2):131–40. doi:10.1016/s0361-9230(00)00415-9 11275401

[45] Woo DH, Bae JY, Nam MH, An H, Ju YH, Won J, Activation of Astrocytic mu-opioid Receptor Elicits Fast Glutamate Release Through TREK-1-Containing K2P Channel in Hippocampal Astrocytes. Front Cell Neurosci. 2018;12:319. doi:10.3389/fncel.2018.00319 30319359 PMC6170663

[46] Woo DH, Han KS, Shim JW, Yoon BE, Kim E, Bae JY, TREK-1 and Best1 channels mediate fast and slow glutamate release in astrocytes upon GPCR activation. Cell. 2012;151(1):25–40. doi:10.1016/j.cell.2012.09.005 23021213

[47] Xie CW, Morrisett RA, Lewis DV. Mu opioid receptor-mediated modulation of synaptic currents in dentate granule cells of rat hippocampus. J Neurophysiol. 1992;68(4):1113–20. doi:10.1152/jn.1992.68.4.1113 1359026

[48] Browne CA, Lucki I. Targeting opioid dysregulation in depression for the development of novel therapeutics. Pharmacol Ther. 2019;201:51–76. doi:10.1016/j.pharmthera.2019.04.009 31051197 PMC6859062

[49] YaDeau JT, Morelli CM, Billingsley JK. Ketamine stimulates secretion of beta-endorphin from amouse pituitary cell line. Reg Anesth Pain Med. 2003;28(1):12–6. doi:10.1053/rapm.2003.50021 12567337

[50] Pacheco Dda F, Romero TR, Duarte ID. Central antinociception induced by ketamine is mediated by endogenous opioids and μ- and δ-opioid receptors. Brain research. 2014;1562:69–75. doi:10.1016/j.brainres.2014.03.026 24675031

[51] Petrocchi JA, de Almeida DL, Paiva-Lima P, Queiroz-Junior C, Caliari MV, Duarte IDG, Peripheral antinociception induced by ketamine is mediated by the endogenous opioid system. European journal of pharmacology. 2019;865:172808. doi:10.1016/j.ejphar.2019.172808 31738939

[52] Tranchand-Bunel D, Delbende C, Guy J, Jegou S, Jenks BJ, Mocaër E, [Pro-opiomelanocortin neuronal systems]. Rev Neurol (Paris). 1987;143(6–7):471–89. 3310184

[53] Veening JG, Gerrits PO, Barendregt HP. Volume transmission of beta-endorphin via the cerebrospinal fluid; a review. Fluids Barriers CNS. 2012;9(1):16. doi:10.1186/2045-8118-9-16 22883598 PMC3439317

[54] Darko DF, Risch SC, Gillin JC, Golshan S. Association of beta-endorphin with specific clinical symptoms of depression. The American journal of psychiatry. 1992;149(9):1162–7. doi:10.1176/ajp.149.9.1162 1503128

[55] Inturrisi CE, Alexopoulos G, Lipman R, Foley K, Rossier J. beta-Endorphin immunoreactivity in the plasma of psychiatric patients receiving electroconvulsive treatment. Ann N Y Acad Sci. 1982;398:413–23. doi:10.1111/j.1749-6632.1982.tb39512.x 6297361

[56] Sacerdote P, Brini A, Mantegazza P, Panerai AE. A role for serotonin and beta-endorphin in the analgesia induced by some tricyclic antidepressant drugs. Pharmacology, biochemistry, and behavior. 1987;26(1):153–8. doi:10.1016/0091-3057(87)90548-x 2951743

[57] Saanijoki T, Tuominen L, Tuulari JJ, Nummenmaa L, Arponen E, Kalliokoski K, Opioid Release after High-Intensity Interval Training in Healthy Human Subjects. Neuropsychopharmacology. 2018;43(2):246–54. doi:10.1038/npp.2017.148 28722022 PMC5729560

[58] Bartlett JD, Close GL, MacLaren DP, Gregson W, Drust B, Morton JP. High-intensity interval running is perceived to be more enjoyable than moderate-intensity continuous exercise: implications for exercise adherence. J Sports Sci. 2011;29(6):547–53. doi:10.1080/02640414.2010.545427 21360405

[59] Zangen A, Nakash R, Yadid G. Serotonin-mediated increases in the extracellular levels of beta-endorphin in the arcuate nucleus and nucleus accumbens: a microdialysis study. Journal of neurochemistry. 1999;73(6):2569–74. doi:10.1046/j.1471-4159.1999.0732569.x 10582620

[60] Deyama S, Bang E, Wohleb ES, Li XY, Kato T, Gerhard DM, Role of Neuronal VEGF Signaling in the Prefrontal Cortex in the Rapid Antidepressant Effects of Ketamine. The American journal of psychiatry. 2019;176(5):388–400. doi:10.1176/appi.ajp.2018.17121368 30606046 PMC6494682

[61] Lepack AE, Fuchikami M, Dwyer JM, Banasr M, Duman RS. BDNF release is required for the behavioral actions of ketamine. Int J Neuropsychopharmacol. 2014;18(1). doi:10.1093/ijnp/pyu033 PMC4368871 25539510

[62] Zhang H, Torregrossa MM, Jutkiewicz EM, Shi YG, Rice KC, Woods JH, Endogenous opioids upregulate brain-derived neurotrophic factor mRNA through delta- and micro-opioid receptors independent of antidepressant-like effects. The European journal of neuroscience. 2006;23(4):984–94. doi:10.1111/j.1460-9568.2006.04621.x 16519663 PMC1462954

[63] Singleton PA, Lingen MW, Fekete MJ, Garcia JG, Moss J. Methylnaltrexone inhibits opiate and VEGFinduced angiogenesis: role of receptor transactivation. Microvasc Res. 2006;72(1–2):3–11. doi:10.1016/j.mvr.2006.04.004 16820176

[64] Jiang C, Lin WJ, Salton SR. Role of a VGF/BDNF/TrkB Autoregulatory Feedback Loop in Rapid-Acting Antidepressant Efficacy. Journal of molecular neuroscience : MN. 2019;68(3):504–9. doi:10.1007/s12031-018-1124-0 30022437 PMC6338529

[65] Hu X, Huang F, Szymusiak M, Liu Y, Wang ZJ. Curcumin attenuates opioid tolerance and dependence by inhibiting Ca2+/calmodulin-dependent protein kinase II alpha activity. The Journal of pharmacology and experimental therapeutics. 2015;352(3):420–8. doi:10.1124/jpet.114.219303 25515789 PMC4352596

[66] Seo YJ, Kwon MS, Choi HW, Jang JE, Lee JK, Jung JS, The differential effect of morphine and beta-endorphin administered intracerebroventricularly on pERK and pCaMK-II expression induced by various nociceptive stimuli in mice brains. Neuropeptides. 2008;42(3):319–30. doi:10.1016/j.npep.2008.01.003 18359081

[67] Banke TG, Bowie D, Lee H, Huganir RL, Schousboe A, Traynelis SF. Control of GluR1 AMPA receptor function by cAMP-dependent protein kinase. J Neurosci. 2000;20(1):89–102. 10627585 10.1523/JNEUROSCI.20-01-00089.2000 PMC6774102

[68] Mammen AL, Kameyama K, Roche KW, Huganir RL. Phosphorylation of the alpha-amino-3-hydroxy-5-methylisoxazole4-propionic acid receptor GluR1 subunit by calcium/calmodulin-dependent kinase II. The Journal of biological chemistry. 1997;272(51):32528–33. doi:10.1074/jbc.272.51.32528 9405465

[69] Esteban JA, Shi SH, Wilson C, Nuriya M, Huganir RL, Malinow R. PKA phosphorylation of AMPA receptor subunits controls synaptic tracking underlying plasticity. Nature neuroscience. 2003;6(2):136–43. doi:10.1038/nn997 12536214

[70] Petraschka M, Li S, Gilbert TL, Westenbroek RE, Bruchas MR, Schreiber S, The absence of endogenous beta-endorphin selectively blocks phosphorylation and desensitization of mu opioid receptors following partial sciatic nerve ligation. Neuroscience. 2007;146(4):1795–807. doi:10.1016/j.neuroscience.2007.03.029 17467916 PMC2012364

[71] Miess E, Gondin AB, Yousuf A, Steinborn R, Mösslein N, Yang Y, Multisite phosphorylation is required for sustained interaction with GRKs and arrestins during rapid μ-opioid receptor desensitization. Sci Signal. 2018;11(539). doi:10.1126/scisignal.aas9609 30018083

[72] Yousuf A, Miess E, Sianati S, Du YP, Schulz S, Christie MJ. Role of Phosphorylation Sites in Desensitization of μ-Opioid Receptor. Molecular pharmacology. 2015;88(4):825–35. doi:10.1124/mol.115.098244 25969388

[73] García-Sevilla JA, Alvaro-Bartolomé M, Díez-Alarcia R, Ramos-Miguel A, Puigdemont D, Pérez V, . Reduced platelet G protein-coupled receptor kinase 2 in major depressive disorder: antidepressant treatment-induced upregulation of GRK2 protein discriminates between responder and non-responder patients. European neuropsychopharmacology : the journal of the European College of Neuropsychopharmacology. 2010;20(10):721–30. doi:10.1016/j.euroneuro.2010.04.008 20493668

[74] García-Sevilla JA, Escribá PV, Ozaita A, La Harpe R, Walzer C, Eytan A, Up-regulation of immunolabeled alpha2A-adrenoceptors, Gi coupling proteins, and regulatory receptor kinases in the prefrontal cortex of depressed suicides. Journal of neurochemistry. 1999;72(1):282–91. doi:10.1046/j.1471-4159.1999.0720282.x 9886080

[75] Grange-Midroit M, García-Sevilla JA, Ferrer-Alcón M, La Harpe R, Huguelet P, Guimón J. Regulation of GRK 2 and 6, beta-arrestin-2 and associated proteins in the prefrontal cortex of drug-free and antidepressant drug-treated subjects with major depression. Brain research. Molecular brain research. 2003;111(1–2):31–41. doi:10.1016/s0169-328x(02)00667-8 12654503

[76] Miralles A, Asensio VJ, García-Sevilla JA. Acute treatment with the cyclic antidepressant desipramine, but not fluoxetine, increases membrane-associated G protein-coupled receptor kinases 2/3 in rat brain. Neuropharmacology. 2002;43(8):1249–57. doi:10.1016/s0028-3908(02)00306-4 12527474

[77] Ponton E, Turecki G, Nagy C. Sex Differences in the Behavioral, Molecular, and Structural Effects of Ketamine Treatment in Depression. Int J Neuropsychopharmacol. 2022;25(1):75–84. doi:10.1093/ijnp/pyab082 34894233 PMC8756094

[78] Aloisi AM, Sacerdote P, Albonetti ME, Carli G. Sex-related effects on behaviour and beta-endorphin of different intensities of formalin pain in rats. Brain research. 1995;699(2):242–9. doi:10.1016/0006-8993(95)00912-a 8616627

[79] Pluchino N, Ninni F, Casarosa E, Giannini A, Merlini S, Cubeddu A, Sex differences in brain and plasma beta-endorphin content following testosterone, dihydrotestosterone and estradiol administration to gonadectomized rats. Neuroendocrinology. 2009;89(4):411–23. doi:10.1159/000209506 19295188

[80] Berrocoso E, Ikeda K, Sora I, Uhl GR, Sánchez-Blázquez P, Mico JA. Active behaviours produced by antidepressants and opioids in the mouse tail suspension test. Int J Neuropsychopharmacol. 2013;16(1):151–62. doi:10.1017/s1461145711001842 22217458

[81] Zomkowski AD, Santos AR, Rodrigues AL. Evidence for the involvement of the opioid system in the agmatine antidepressant-like effect in the forced swimming test. Neuroscience letters. 2005;381(3):279–83. doi:10.1016/j.neulet.2005.02.026 15896484

[82] Broom DC, Jutkiewicz EM, Folk JE, Traynor JR, Rice KC, Woods JH. Nonpeptidic delta-opioid receptor agonists reduce immobility in the forced swim assay in rats. Neuropsychopharmacology. 2002;26(6):744–55. doi:10.1016/s0893-133x(01)00413-4 12007745

[83] Gerner RH, Catlin DH, Gorelick DA, Hui KK, Li CH. beta-Endorphin. Intravenous infusion causes behavioral change in psychiatric inpatients. Archives of general psychiatry. 1980;37(6):642–7. doi:10.1001/archpsyc.1980.01780190040005 7387336

[84] Kline NS, Li CH, Lehmann HE, Lajtha A, Laski E, Cooper T. Beta-endorphin--induced changes in schizophrenic and depressed patients. Archives of general psychiatry. 1977;34(9):1111–3. doi:10.1001/archpsyc.1977.01770210125012 901140

[85] Pickar D, Davis GC, Schulz SC, Extein I, Wagner R, Naber D, Behavioral and biological effects of acute beta-endorphin injection in schizophrenic and depressed patients. The American journal of psychiatry. 1981;138(2):160–6. doi:10.1176/ajp.138.2.160 6257125

[86] Nibuya M, Morinobu S, Duman RS. Regulation of BDNF and trkB mRNA in rat brain by chronic electroconvulsive seizure and antidepressant drug treatments. J Neurosci. 1995;15(11):7539–47. doi:10.1523/jneurosci.15-11-07539.1995 7472505 PMC6578063

[87] Choi M, Lee SH, Park MH, Kim YS, Son H. Ketamine induces brain-derived neurotrophic factor expression via phosphorylation of histone deacetylase 5 in rats. Biochem Biophys Res Commun. 2017;489(4):420–5. doi:10.1016/j.bbrc.2017.05.157 28577999

[88] Zhang XY, Dou YN, Yuan L, Li Q, Zhu YJ, Wang M, Different neuronal populations mediate inflammatory pain analgesia by exogenous and endogenous opioids. Elife. 2020;9. doi:10.7554/eLife.55289 PMC7311172 32519950

